# “We are caring for the whole person”: A qualitative study of social work’s role in palliative cancer care

**DOI:** 10.1017/S1478951525101466

**Published:** 2026-01-16

**Authors:** Ting Guan, Arden O’Donnell, Shameem Varikkodan, Sadaf Sedaghatshoar, Karlynn BrintzenhofeSzoc

**Affiliations:** 1 School of Social Work, Indiana University, Indianapolis, IN, USA; 2 Melvin and Bren Simon Comprehensive Cancer Center, Indiana University, Indianapolis, IN, USA; 3 School of Social Work, Boston University, Boston, MA, USA; 4 Kent School of Social Work and Family Science, University of Louisville, Louisville, KY, USA

**Keywords:** Role, social work, palliative care, cancer, job description

## Abstract

**Objectives:**

The importance of palliative care in cancer care is underscored, yet there is a significant gap in research specifically focusing on the role of social workers in palliative cancer care. This qualitative study aims to better articulate the specific roles of social workers within palliative oncology settings.

**Methods:**

Data were collected by semi-structured Zoom interviews with social workers in palliative cancer care between November 2023 and January 2024. Thematic analysis was used to identify unique themes.

**Results:**

Ten social workers in palliative cancer care were recruited for this study. Eight key themes related to social workers’ role emerged from the interviews. These were the following: (1) mapping out holistic needs through a biopsychosocial–spiritual assessment, (2) providing individual and family counseling, (3) patient and family psychoeducation, (4) resource identification and referral, (5) building communication bridges between patients, families, and oncology teams, (6) promoting patient and family engagement and voice in shared decision-making in cancer care, (7) providing anticipatory grief and bereavement counseling, and (8) strengthening team resilience and fostering well-being.

**Significance of results:**

This study builds upon prior work by focusing specifically on the roles of palliative care social workers in oncology. The findings highlight the multifaceted roles of social workers, demonstrating their capacity to deliver holistic care to cancer patients, families, and healthcare providers to enhance quality of care. The findings may help inform the development of training curricula and practice standards for the subspecialty of oncology-focused palliative social work.

## Introduction

Over the past decades, palliative social work in the United States has evolved into a specialty, evidenced by education programs and a professional certification (Csikai and Bullock 2024). As the primary providers of psychosocial care, social workers are integral members of the palliative care team, with 68% of palliative care programs in the United States having a social worker on their teams (Rogers and Heitner 2019). Rooted in a strong theoretical foundation – including psychodynamic theory, family systems theory, and social ecological model – social workers in palliative care offer cursory emotional support and provide resource referrals (Terry Altilio and Otis-Green 2011). Their essential role is underscored in national guidelines such as the *Clinical Practice Guidelines for Quality Palliative Care*, which name social workers as leaders in addressing psychological, cultural, and social domains of care (National Consensus Project for Quality Palliative Care 2018). Empirical studies underscore the essential contributions of palliative social workers in enhancing patient-centered quality of care. When social workers lead or are integrated into palliative care services, patients are more likely to have their psychosocial needs addressed, including psychological, social, and spiritual concerns, advance care planning, and goals-of-care discussions (O’Donnell et al. 2018; Edmonds et al. 2021).

Patients with cancer represent a significant portion of individuals with serious illness (Alnajar et al. 2025). The origins of palliative care are closely tied to the care of people with cancer (Clark 2007). Similar to general palliative care, palliative care in oncology offers a holistic approach that emphasizes symptom management, psychosocial support, and assistance with decision-making, with the goal of improving the quality of life for patients with cancer and their families. In contrast to patients with other serious illnesses (e.g., heart failure and chronic obstructive pulmonary disease), who often experience unpredictable disease courses, patients with cancer typically follow a more predictable trajectory (Steinhauser et al. 2011) with complex and evolving needs (Bandeali et al. 2020). This trajectory is often characterized by periods of relative stability, opportunities for additional treatment, and the possibility of a sudden transition to end-of-life care (Smith et al. 2022). Palliative care may be initiated for patients with cancer at any point in the illness trajectory, from diagnosis to the end of life, while they continue to receive active treatment (Smith et al. 2012). This model of care depends on close collaboration between oncology clinicians and palliative care specialists (Hui et al. 2015). Accumulating evidence suggests that early integration of palliative care in oncology is associated with improved quality of life (Bakitas et al. 2009), better psychological outcomes (El-Jawahri et al. 2021), and greater satisfaction with care (Zimmermann et al. 2014). In recognition of these positive outcomes, there have been recent calls to advance palliative care in oncology. The American Society of Clinical Oncology (ASCO) has published guidelines advocating for early integration of palliative care into usual oncology practice and highlighting the need for workforce development to meet the growing demand for palliative cancer care (Ferrell et al. 2017; Sanders et al. 2024).

In response to the growing emphasis on palliative care as an integral part of oncology, social workers also need to enhance their specialization to remain relevant in the field. Although several studies have proposed core competencies for social workers in palliative and end-of-life care (Gwyther et al. 2005), generalist-level palliative care (Glajchen et al. 2018), and pediatric palliative care (Jonas et al. 2022), much of this literature is based on expert opinion and conducted within broad palliative care contexts (Head et al. 2019). There remains a significant gap in research focusing on the role of social workers in palliative cancer care (Guan et al. 2024). To address this gap, this qualitative study aims to better articulate the specific roles of social workers within palliative oncology settings. The findings aim to inform practice standards, interdisciplinary training, and the further development of oncology-specific palliative social work competencies.

## Methods

This qualitative study employed semi-structured interviews via Zoom to explore social workers’ roles when engaging in palliative care practice with the patients with cancer and their families. This study was approved by Syracuse University Institutional Review Board (# 23-266).

### Recruitment and data collection

Participants were recruited through convenience sampling. Inclusion criteria included: (1) social workers currently practicing with cancer patients in the United States and (2) engagement in the provision of palliative care. Recruitment was conducted through an announcement sent by a national Listserv managed by Social Work Network in Palliative and End-of-Life Care. A recruitment flyer, which included a request for sharing it with other social workers, was sent to a local cancer setting. Interested social workers completed a screening survey in Qualtrics to determine eligibility. Those who were eligible were contacted by a member of the research team via email to arrange an interview and obtain further confidential demographic information.

All participants provided informed oral consent before participating in the interview. Prior to each interview, the interviewers introduced themselves, disclosed their personal and professional positionality, and described the aims of the study. An interview guide (Supplementary material 1) was developed based on the research questions and a review of the literature. The interview questions included participants’ current job and position, their experience in palliative cancer care, interdisciplinary collaboration, and the challenges they encountered. The interviews were conducted via Zoom by the first author (T.G.), a female researcher with a PhD in social work, or by a trained female master’s-level research assistant with training in qualitative research methods. After each interview, the interviewers wrote field notes, including interview summaries. The interviews were audio- or video-recorded and lasted from 18 to 43 minutes (median 32 minutes). The participants were compensated with a $30 Amazon eGift card upon completion. Data were collected between November 2023 and January 2024.

### Data analysis

The audio recordings were initially transcribed using Zoom’s automatic transcription feature and subsequently cleaned and verified for accuracy by 4 authors (T.G., S.V., S.S., and K.B.). The transcripts were analyzed in Word documents, guided by reflexive thematic analysis (Braun and Clarke 2006; Braun and Clarke 2024). Two authors (T.G. and K.B.) developed a codebook based on a preliminary review of the 2 pilot transcripts, and coding was conducted inductively from the data. Four authors (T.G., S.V., S.S., and K.B.) worked independently on formal coding. Regular team meetings were conducted to resolve discrepancies. Once the initial codes were generated, we categorized and combined them to develop potential themes. The entire team then collaboratively reviewed and refined the themes through critical dialogue, moving iteratively between selected extracts from the full dataset to ensure that the themes accurately reflected the data and captured its breadth and depth.

### Researcher characteristics and reflexivity

Two research team members had prior clinical experience in palliative care and cancer care in the United States, while 3 others had international backgrounds in social work practice. Throughout the process of code and theme development, the team engaged in careful reflection and explicitly acknowledged their own positionality. Reflexivity was intentionally applied to reduce bias and to ensure that prior knowledge did not interfere with generating new insights from the data.

## Findings

### Participants

Ten social workers in palliative cancer care were recruited. Participants had been in their roles for between 2 and 40 years, with 8 working as clinical social workers and 2 holding supervisory roles. Eight participants provided demographic information. Sixty-three percent of the participants were over 50 years old, predominantly female (88%) and White (63%). Additionally, 88% held full licenses (e.g., Licensed Master Social Worker, Licensed Clinical Social Worker) and 25% reported having advanced palliative and hospice social work certification (APHSW-C).

### Social workers’ role in palliative cancer care

Eight key themes related to social workers’ roles emerged from the interviews. These were the following: (1) mapping out holistic needs through a biopsychosocial–spiritual assessment, (2) providing individual and family counseling, (3) patient and family psychoeducation, (4) resource identification and referral, (5) building communication bridges between patients, families, and oncology teams, (6) promoting patient and family engagement and voice in shared decision-making in cancer care, (7) providing anticipatory grief and bereavement counseling, and (8) strengthening team resilience and fostering well-being.

#### Theme 1. Mapping out holistic needs through a biopsychosocial–spiritual assessment

Participants emphasized the importance of identifying unmet needs through comprehensive biopsychosocial–spiritual assessments conducted during their initial visit. These holistic assessments inform and guide social workers’ interventions:

I see every patient that comes into our clinic on their first visit, and I complete a psychosocial assessment to determine if there’s any unmet needs.

We assess how they’re doing and how we may best help them in sort of the mind, body, spirit adjustment to having a serious illness.

Social workers cited various theoretical frameworks guiding their assessments. For example, 1 participant who worked with adolescents and young adults described adjusting the focus of psychosocial assessments based on the developmental needs of patients (sexual health, substance use, and family and social relationships):

[With patients] ages 18 to 39 who are diagnosed with any type of cancer, biopsychosocial assessments [are done] … with a focus on elements of their personhood and health and interactions with their cancer care that are related to their developmental stage …Focusing on things like sexual health and use of like alcohol and other substances, and like dating … having young children, being parents, jobs, school.

The biggest part of my role is emotional support, crisis intervention [with] cancer patients who are diagnosed with terminal cancer.

Social workers also cited using validated screening tools to assess distress, depression, anxiety, and suicide ideation to identify areas of vulnerability and need:

We do distress screening; sort of, the initial assessment is using the NCCN (National Comprehensive Cancer Network) distress screening, which is a cancer tool and that helps us direct a little bit about the plan of care psychosocially that patients need.

When I first meet a patient, I do the full Columbia assessment, and then every follow up appointment I do the brief 4 or 3 question Columbia [Columbia-Suicide Severity Rating Scale], and the PHQ-9 [Patient Health Questionnaire] and the GAD-7 [General Anxiety Disorder-7].

In addition, social workers were acutely aware of the effect of trauma on an individual’s physical and mental health, particularly with advanced illness. Social workers articulated using a trauma-informed care lens to provide tailored support for the patient and to guide the team to create a safe and responsive environment:

We ask about childhood trauma, or even trauma as an adult, and how we might be mindful of that when we care for them. If I know that there’s that link to a traumatic history … [it] helps me communicate to my team members because a person may be behaving a certain way. It’s all from a trauma informed lens …how can I and my colleagues create safety knowing that this person has this history. Do we need to be more mindful about the way that we approach it? Do we need to be more accepting of their hesitation to engage.

#### Theme 2. Providing individual and family counseling

Participants cited providing targeted counseling for both patients and their family members. This counseling was tailored to address concerns identified during the assessment, such as pain management, anxiety, depression, and family relationships. The format (individual, couple, or family), structure, and duration of the counseling sessions varied based on patients’ and families’ needs and circumstances:

I do a lot of one-on-one counseling with the patients themselves. That’s a big part of my role. Our clinic is very focused on also counseling the care partners to anyone who may be living with the illness themselves. - Sometimes we’ll do family counseling or couples counseling, that’s the biggest part of my role is that emotional support and counseling around any serious illness-related stressors.

Often those are followed up by like hour long counseling sessions that are scheduled with me, or I’ll do sort of kind of less structured counseling. Depending on the circumstances of where people are physically in the hospital and what’s going on and how they’re feeling physically.

Social workers spoke of incorporating various evidence-based counseling techniques into their sessions, citing meaning-centered therapy, motivational interviewing, strengths-based therapy, Acceptance and Commitment Therapy (ACT), and Cognitive Behavioral Therapy (CBT):

My counseling work tends to focus on aspects of like meaning making and illness, particularly with serious illness…my counseling work tends to focus on impacts of having a serious illness on people’s relationships and on their sort of goals, aspirations and life, other life circumstances.

I’m generally doing strengths-based interactions and techniques with them. But when it’s necessary I’ll use motivational interviewing. I’ll use ACT. I’ll use a CBT, I’m trained in CBT for insomnia, and so I’ll use that if it’s necessary. So it kind of depends what is needed for a patient.

#### Theme 3. Patient and family psychoeducation

Participants provided psychoeducation for patients and their families. For example, they provided patients with information about fertility preservation options and risks, as well as healthcare benefits related to hospice care:

We have another social worker who, primarily, she directs our fertility preservation program. She does really critical work and educating people about fertility risks and fertility preservation options and helping them through that process.

Sometimes just providing that explanation of the Hospice benefit [helps] a lot, I mean the actual hospice benefit, the benefit under Medicare.

#### Theme 4. Resource identification and referral

Social workers described a central aspect of their role as facilitating linkages between the healthcare system and broader human service systems in order to address patients’ and families’ social needs. These needs commonly included access to food, housing, transportation, and financial support. One participant noted:

We also talk about the social determinants of health. We also provide the social resource support for them.

Another social worker highlighted the proactive search for resources:

[I] always look for additional resources. Either with nonprofit cancer organizations in our community or donors to our cancer center to think about opportunities of how we can use, like paying for Ubers for them to get to treatment or getting them food delivery services.

In addition to linking patients with resources, participants described their roles as extending to care coordination and advocacy, ensuring that patients and families receive comprehensive support across healthcare and social service systems.

#### Theme 5. Building communication bridges between patients, families, and oncology teams

Participants emphasized their role as communication bridges, ensuring that patients’ and families’ concerns, goals, and preferences were conveyed to oncology providers. They described serving as the “link” between patients and the medical team:

A lot of times our role is that communicator. I know what that patient and family are worried about. I then link the oncologist into that communication. So sometimes we’re the communication link.

This communication role extended to timely updates, especially when urgent issues or goals-of-care discussions arose. Social workers described proactively reaching out to oncologists to ensure alignment across the care team:

If it’s something more urgent, just letting [primary oncologists] know we’ve kind of had a discussion about X, Y, and Z goals, and wanting them to be aware.

Participants also highlighted medical records as practical tools for bidirectional communication:

I think we have a great medical record that makes it really easy to communicate with the oncology team. For most oncologists, it’s not abnormal for me to reach out and say, “I’m meeting with this person, I wanted to share my note with you” – and vice versa. We might get updates from the oncology team: “We just saw this patient, this is what’s happening, be aware, or we’d love some support around this specific issue.”

Through these practices, social workers played a pivotal role in strengthening communication channels, fostering collaboration, and ensuring coordinated cancer care.

#### Theme 6. Promoting patient and family engagement and voice in shared decision-making in cancer care

Participants felt they created a unique, empathic “space” for patients and families to explore their goals and values, allowing them to process the emotional impact of illness and to make informed decisions that align with patient values:

I can provide a space for people to be able to acknowledge those things that they otherwise are sort of having to worry about alone, or to just deal with internally and that ideally providing that space also can lead to people being able to make informed choices whether that’s choices about their care, choices about what they want to do with their time choices about how they want to live and achieve the best quality of life they can.

A central aspect of this role involved centering the patient’s voice, advocating for their needs and preferences, and bridging communication between patients and providers. Social workers emphasized their role in mediating conflicts and facilitating mutual understanding:

I do a lot of helping patients learn how to advocate for their own needs and their own medical decision-making preferences with their providers. I do a lot of working with their providers to help them understand their patients, and where their patients are coming from, and bridging communication gaps and mediating conflict between patients and their providers, and often families are in the mix somewhere in there as well.

Social workers cited using family meetings as a platform to share medical information, discuss treatment options, and provide emotional support. Participants noted that a key social work role was facilitating family meetings to clarify goals of care and end-of-life plans with the whole team:

Coordinating that family meeting to discuss that this journey is coming to an end and give the family an opportunity to discuss what they wanted that end to look like because a tenant that I work [by is that] every patient is entitled to a good death …. That was serving this patient with most dignity and honor and respect.

The participants also facilitated advance care planning, such as healthcare proxies, and end-of-life discussions:

I have end of life discussions, advance care planning discussions, so talking to them about the importance of having healthcare proxy. I might give them a form like the 5 Wishes and then talk to them about it. Because I think it helps them to think about how they want things to be going forward.

#### Theme 7. Providing anticipatory grief and bereavement counseling

Patients and their family members often experience anticipatory grief and secondary losses related to cancer even before the patient’s death. Participants described using various grief frameworks, such as the dual process model of coping with bereavement, to help patients, couples, and families process ongoing losses and validate their emotional experiences through therapeutic dialogue. They employed specific counseling techniques to address grief-related needs, particularly in navigating anticipatory grief:

I think a lot of it for the family members is it’s a lot of counseling about a lot of anxiety and grief related to secondary losses and their expectations for the future. A lot of grief counseling, even though the person hasn’t died yet.

I think a lot of different ways to conceptualize grief, grief frameworks, bringing that into the therapeutic space and talking about it. [Like] the dual process model of coping with bereavement. I think it’s so helpful to show that to an individual or a couple or a family and it’s actually real that you would be spending some time thinking about all the losses that are happening in real time.

They also provided and coordinated bereavement services for family members:

A common resource that I provide to patients with late-stage cancer is a local bereavement organization for children. And that’s usually for children whose parents are the patients with cancer and are dying.

#### Theme 8. Strengthening team resilience and fostering well-being

Social workers not only provided holistic care to cancer patients but also attended to the emotional needs of the entire palliative care team. They supported their colleagues through clinical debriefing and reflective practices, especially when they faced difficult cases:

Supporting our colleagues is also important. Educating the nurse partitioner on my team and I meet regularly with the new nurse residents who are in their first year of employment. And we do a clinical debriefing with them. So again support them in doing some hard work and facing tough issues.

Several participants also identified themselves as key team members responsible for fostering team well-being. For instance, they offered emotional support to colleagues experiencing moral distress. As 1 social worker shared:

As teams experience distress or bereavement or just you know, some suffering as they watch patients deteriorate. I think social workers are often the people who provide the emotional support not only for patients and families, but for teams as well.

## Discussion

This study builds on prior work by focusing specifically on the roles of palliative social workers in oncology. The findings highlight the multifaceted roles of social workers, demonstrating how social workers deliver holistic care to patients with cancer, their families, and healthcare teams. These roles align with core areas outlined in the National Association of Social Workers (NASW) published Practice Standards for Palliative and End of Life Care (National Association of Social Workers n.d.) and the National Consensus Project Clinical Practice Guidelines for Quality Palliative Care (National Consensus Project for Quality Palliative Care 2018). Moreover, the findings highlight the unique roles of social workers in palliative cancer care, including facilitating communication between oncology and palliative care teams, supporting informed medical decision-making, and promoting dialogue among patients, families, and clinicians.

The holistic biopsychosocial–spiritual assessment represents a central responsibility of social workers in palliative cancer care. For many patients with cancer and their families, the physical burden of illness is compounded by psychological distress. Research indicates that nearly one-third of patients in palliative care settings experience depression, anxiety, and/or adjustment disorder (Mitchell et al. 2011). Accordingly, social workers play a critical role in assessing common psychological issues using clinically rigorous and validated tools. Beyond psychological concerns, patients with cancer frequently experience diverse and complex needs across physical, psychosocial, informational, financial, fertility, sexual, spiritual, and relational domains. Social workers are uniquely positioned to identify these unmet needs through comprehensive biopsychosocial–spiritual assessments, often conducted during initial encounters, and to ensure that psychosocial services are delivered – either directly, in consultation, or through referral to other providers.

Social workers in palliative oncology are well trained and positioned to address the psychological needs of cancer patients, drawing on the distinct skills and competencies inherent to the profession. Though not directly prompted, social workers cited various theories as guiding frameworks. Equally important, they emphasized the necessity of evidence-based supportive practices that are not only theory-informed but also adapted to the local contexts and lived realities of those receiving care. In practice, social workers reported integrating a range of evidence-based approaches into their services, such as meaning-centered therapy, motivational interviewing, strengths-based therapy, ACT, and CBT. In addition to these practices, social workers provide up-to-date supportive care programs for patients with cancer. Emerging research underscores the cumulative impact of trauma, both across the lifespan and throughout the cancer care continuum (Davidson et al. 2023; Fulton et al. 2024). In this context, social workers are uniquely positioned to deliver trauma-informed care within palliative settings.

Within the context of palliative oncology, social workers play a critical role in facilitating informed medical decision-making and promoting communication among patients, families, and clinicians. Advances in treatment have enabled many patients with advanced cancer to live longer, bringing goals of care to the forefront. Even when diagnosed with advanced or metastatic cancer, patients remain actively engaged in their cancer care and survivorship planning. Compared with individuals affected by other serious illnesses, a relatively larger proportion of patients with cancer report substantial decision-making needs, particularly in relation to treatment options and goals of care (Shah et al. 2025). Social workers’ active involvement is essential to ensuring that the preferences, values, and personal agency of patients and their families are respected and supported. A collaborative shared decision-making framework not only fosters alignment with patients’ individual goals but also strengthens the overall quality of care provision (Kane et al. 2014). Furthermore, the relative predictability of cancer trajectories allows for more deliberate planning and preparation for end-of-life care compared to many other life-limiting conditions. Our findings underscore the central role of social workers in leading advance care planning discussions, coordinating and participating in family meetings, and evaluating the cultural, social, and relational contexts that shape patients’ and families’ experiences.

Patients with cancer, particularly those with advanced disease, often experience multiple care transitions across different healthcare settings and providers. One critical transition occurs when patients move from oncology to palliative care teams. In the current co-management model (Jacobsen et al. 2016), palliative care teams bring the expertise to help patients, and their families manage symptoms, clarify goals of care, and make informed treatment decisions, while oncology clinicians focus on directing cancer therapy, stabilizing disease, and prolonging life. Social workers play a pivotal role in bridging these two models to ensure continuity of health services across survivorship and palliative care phases.

Finally, drawing on their deep knowledge of community resources, social workers help patients and families navigate, and access a wide range of services, from insurance benefits to community support services. These mezzo-level social work practices include working to remove barriers and advocating for needed services to create healthcare environments that enhance their patients’ and family members’ functioning. As social workers are uniquely positioned to address healthcare disparities and health equity using a social justice framework (Ashcroft et al. 2021), they not only fulfill a central focus of palliative care but also have the skills to practice macro social work such as advocating to minimize the barriers rooted in social determinants of health.

Beyond patient care, social workers also address the emotional well-being of the broader palliative care team. This study affirms the value of social workers in supporting team functioning, underscoring their role not only as clinicians but also as essential contributors to healthy team dynamics. They contribute to team cohesion and resilience by offering informal debriefing and actively engaging in reflective practice to process their own emotional responses, and modeling emotional resilience to foster psychological safety among colleagues. The NASW practice standards emphasize cultural competence as a foundational requirement for social workers (National Association of Social Workers n.d.). Our findings suggest that social workers play a critical role in integrating cultural humility into the interdisciplinary care team, ensuring care plans respect and incorporate patients’ cultural values and beliefs.

This study extends existing literature by offering more in-depth insight into the roles of social workers in palliative cancer care. The findings can be used to inform the training curricula and practice standards for the subspecialty of oncology-focused palliative social work. Limitations include the lack of intentional comparison of social workers’ roles across settings (inpatient versus outpatient), which may obscure important differences in practice. Future research could explore role distinctions and required skills across different palliative care contexts, as well as the broader contributions to advancing population health and reducing healthcare costs. In doing so, the field can move toward formalized training pathways and competency standards that reflect the depth and impact of social work in serious illness care.

## Conclusions

As the significance of palliative care in oncology continues to grow, it is essential to recognize the holistic care and unique skills that social workers contribute to this important field. The findings highlight the multifaceted roles of social workers, demonstrating their capacity to deliver holistic care to cancer patients, families, and healthcare providers to enhance quality of care. These insights can inform the development of training curricula and practice standards for the subspecialty of oncology-focused palliative social work, with the ultimate goal of enhancing social workers’ roles in delivering high-quality, patient-, and family-centered care.

## Supporting information

10.1017/S1478951525101466.sm001 Guan et al. supplementary material 1 Guan et al. supplementary material

10.1017/S1478951525101466.sm002 Guan et al. supplementary material 2 Guan et al. supplementary material

## Data Availability

The data are currently used for other studies and manuscript development. The data that support the study may be available upon request with permission from the researchers who collected the data.

## Supplementary material

The supplementary material for this article can be found at https://doi.org/10.1017/S1478951525101466.
