# Supplementary material for: “We are caring for the whole person”: A qualitative study of social work’s role in palliative cancer care
Source: Palliat Support Care. 2026 Jan 16;24:e36. doi: 10.1017/S1478951525101466 (PMC13166357; doi:10.1017/S1478951525101466)
Supplement: Guan et al. supplementary material 1 — Guan et al. supplementary material [file S1478951525101466sup001.docx]

**Interview Question Guide**

1. How long have you been working as a social worker in palliative care in cancer settings?
2. Please describe your job or role as a social worker with patients with cancer receiving palliative care. (Can you give some examples of the kinds of tasks you do when working with patients with cancer).
3. At what points do you provide social work services to patients? For example, at first

diagnosis, during treatments, or during end of life care?

1. How are these cancer patients referred to you?
2. In what ways do social workers in your setting contribute to the delivery of comprehensive care for patients with cancer and their families? Please give any examples you can.
3. How do you collaborate with other health care providers, such as oncologists, palliative care physicians, nurses, and chaplain?
4. How do you collaborate with other social workers, such as medical social workers or community social workers?
5. What are some of the challenges you face as a social worker in your practice setting (personal barriers, institutional barriers). How have you overcome these challenges? What are a few examples?
6. Have there been situations where you have felt your social work services would have been beneficial, but they were not called upon? If yes, can you describe the situation? What services do you think you could have provided in that situation?

Is there anything else you would like to tell us about your role as a social worker that you have not expressed during this interview? If yes, please explain.

**Thank you for participation in this interview.**
