# Supplementary material for: “We are caring for the whole person”: A qualitative study of social work’s role in palliative cancer care
Source: Palliat Support Care. 2026 Jan 16;24:e36. doi: 10.1017/S1478951525101466 (PMC13166357; doi:10.1017/S1478951525101466)
Supplement: Guan et al. supplementary material 2 — Guan et al. supplementary material [file S1478951525101466sup002.docx]

**Code, Definition/Description, and Examples**

| **Code** | **Definition /Description** | **Example** |
| --- | --- | --- |
| Working year | Number of years working in social work | I’ve been in this field for 10 years. |
| Serve population | Primary patient populations served, such as adults, pediatric, hematology/oncology | I'm an adolescent and young adult oncology, clinical social worker. But I've started to redefine my role as adolescent young adult palliative care oncology, social worker. |
| Setting | Practice setting, such as inpatient, outpatient, other | I am only working in the outpatient setting. |
| Education | Degrees held, such as MSW, DSW/PhD | I have a master's in social work. |
| License | Professional licensure, such as LSW, LMSW, LCSW | I have a licensed clinical social worker. |
| Certification | Specialty credentials, such as oncology social work certification, palliative care and hospice certification | I also am a certified Palliative Hospice social worker. |
| Biopsychosocial assessment | Conduct holistic assessments of patient needs (emotional, social, financial, etc.) | I'll do a psychosocial assessment. So just kind of if, unless I've seen them before, but you know I'll do what I would normally do with any cancer patient which is to gather. You know, a little bit of history. |
| Counseling | A structured, therapeutic process in which the social worker engages individuals, families, or groups | Often those are followed up by like hour, long counselling sessions that are scheduled with me, or I'll do sort of kind of less structured counseling. That sometimes is very brief, sometimes is longer. |
| Family support | Emotional and practical support for family caregivers | We do recognize caregivers as our patients too. So a spouse can call, a daughter can call. We can do counselling with them. We can just sort of meet them where they are and help them with their journey. |
| Resource identification and referrals | Connect patients to practical assistant | We also provide any other like resources or the social support, like other like community resources for them. |
| Advocacy | Support patient rights, needs, and preferences | But I do a lot of helping patients learn how to advocate, for their own needs and their own sort of like medical decision-making preferences with their providers. |
| Facilitate decision-making | Help patients/caregivers navigate complex decisions | One of the main things that I feel like I can provide is just a space for people to be able to acknowledge those things that they otherwise are sort of having to worry about alone, or to just deal with internally and that ideally providing that space also can lead to people being able to make informed choices whether that's choices about their care, choices about what they want to do with their time choices about how they want to live and achieve the best quality of life they can, choices about doing things like legacy work or advanced care planning, or wills, or all of that kind of stuff. |
| Facicite communication between patients and health provider | Support communication between patients, caregivers, and medical providers | I do a lot of working with their providers to help them understand their patients, and where their patients are coming from, and bridging communication gaps and mediating conflict between patients and their providers. |
| End of life care | Help patients create meaningful end-of-life expressions, such as advance care planning, advance directives, healthcare proxy | I have end of life discussions, advance care planning discussions, so talking to them about the importance of having healthcare proxy, |
| Grief and bereavement | Support families before and after patient death | I think a lot of it is that, like the different grief, the ways to conceptualize grief, grief frameworks, bringing that into the therapeutic space and talking about it, whether it's kind of like there's this, there's this. You may be familiar with it, but it's the dual process model of coping with bereavement. Right? It's a really, I think it's so helpful to be to show that to an individual or a couple or a family and be like it's actually real that you would be spending some time thinking about all the losses that are happening in real time, and you might be oscillating to that other domain where, you know, I'm evolving with this illness, right? And that's a framework that we can talk about. And they can. I think, therapeutically, it's the idea that 2 things are happening, and 2 things can be true. I can be feeling really sad and being Having grief or anger, frustration, I can also be. You know, there might be things where, like we are, feels weird to say it, but like growing or changing or adapting to this hardship that's come into our life as well. |
| Patient psychoeducation | Provide information about coping, resources, treatment side effects, etc | You know, I'm a social worker with our AYA program. We have another social worker who actually, primarily, she directs our fertility preservation program. So she does really critical work and educating people about fertility risks and fertility preservation options and helping them through that process. |
| Family meetings | A structured conversation involving patients, family members, and healthcare providers to discuss diagnosis, treatment, goals of care, and support needs | Well, if I'm getting a consult for end-of-life care, coordinate a family meeting. So we're working with both the in house team hospitalist as well as the treating team, medical oncologist or surgical oncologist to bring the family together to say, here's where we are. Don't think further treatment is recommended. |
| Team well-being | Enhance the emotional health, morale, and functioning of the healthcare team | As teams experience distress or bereavement or just you know, some suffering as they watch patients deteriorate. I think social workers are often the people who provide the emotional support not only for patients and families, but for teams as well. And I also think that social workers try to bring more culturally curious lens to things. And I think that's so much a part of our training that is different and distinct from some of our counterparts. |
| Intervention timepoints | When social work services occur (diagnosis, active treatment, recurrence, survivorship, end-of-life) | we also have worked with patients. With a new diagnosis at a low stage of cancer as well as new diagnosis with stage 4 cancer. |
| Referral method | How patients or families are connected to social work services, including formal processes (e.g., screening, provider orders) and informal processes (e.g., staff recommendation or self-referral) | And then we also, I get messages just from individual providers. asking me to see someone or re-engage with someone. I do get messages from patients sometimes that I've like already met, but then kind of haven't necessarily followed consistently, cause they didn't need that at the time. |
| Interdisciplinary Collaboration | Collaboration with a broad medical team member, including palliative care physician, nurses, oncologists | I certainly am like collaborating with multidisciplinary providers like all day every day. So I mean, probably that's just within my team, my [maybe I am] co-leader of clinical service with a nurse practitioner but and we have a few physicians on our team as well that I work closely with. |
| Intradisciplinary Collaboration | Collaboration with other social workers within and outside the institution | She knows a lot more people in the community because patients would get transferred to the hospital and things like that. So she does sometimes collaborate with the other social workers, or call them up and sort of pick their brains about things. |
| Social work contribution | How social work contributes to patient care, team functioning, or hospital system | We try to treat the whole person. psychologically and emotionally, people are devastated by a cancer diagnosis. And certainly, if they're, you know, a stage for cancer patient, they're really, you know, and they just, you know, they just have discovered that. And they're in palliative care. I mean, I think that's a terrible time for them. They're really struggling emotionally. So, yeah, I think we we play a really important role at that time |
| Societal/policy facilitator | Societal/policy Factors that support effective social work practice | And there's kind of a movement towards home based palliative care support, and expanding that, but not being siloed from the institution and from the treating team. |
| Organizational Facilitator | Organizational factors that support effective social work practice | Social worker [name] has come on since she came from that nursing, home setting and assisted living setting. she knows a lot more people in the community because patients would get transferred to the hospital and things like that. So she does sometimes collaborate with the other social workers, or call them up and sort of pick their brains about things. |
| Interpersonal facilitator | Interpersonal factors (Relationships and social networks: family, friends, peers, caregivers, coworkers) that support social work practice | We have a collaborative between me and her [palliative care physician] and the sarcoma oncologist who’s also a member of our team as our medical oncology liaison… Anyway, we have a young adult palliative care–sarcoma collaborative. |
| Societal/policy barrier | Societal/policy challenges (Broader cultural, economic, and political influences: law, policies, cultural norms, systemic inequities) that hinder social work practice | I think it is a challenge that (Pause) I think a lot of people face in sort of normalizing, palliative care for patients and families. And I think that can be particularly challenging with young adults. because of sort of the stigmas and perceptions of palliative care. |
| Organizational barrier | Organizational challenges that hinder social work practice | I think institutionally, it would be space and logistics. We are like on the other side of our property from the cancer center in the hospital and everything. We see patients in our clinic, and that's just fine, even though nobody knows where to go at first. Easy but then, when we want to go see patients in the cancer center, it's a little bit of a hike, and then we have to come back here when we have our patients who we see appointments for in the clinic and we have to meet all of these rigid demands…We can't dictate the scheduling of about these patients, you know. They just see this patient in the cancer center, and then within 5 min after you see them, you have to be back here to see these patients. |
| Interpersonal barrier | Interpersonal challenges (Relationships and social networks: family, friends, peers, caregivers, coworkers) that hinder social work practice | I think they have a hard time understanding. I think everyone across the board, even patients, have a hard time understanding what we do as social workers. I think their belief is that we are case managers, and that we, you know, we're in the world to steal their children and put them on food stamps. I don't know. |
| Individual barrier | Individual challenges (Personal characteristics: knowledge, attitudes, skills, genetics, mental health, personality) that hinder social work practice | So there was a really steep learning curve for me to learn how to do the job, and it took me 2 and a half years to really feel like I was doing it. |
| Patient unmet needs | Aspects of care, support, or information that patients feel are missing or insufficient | If our services had been called on earlier. There's a lot of stuff that I think my colleagues could have addressed more robustly than maybe did have. I'm ho! I hope that fertility and sexual health and things like that have been addressed. But I don't know, |
| Suggestions | Ideas or recommendations for enhancing social work services | Tell them what the process will be like, and you know, just make them more comfortable and give them a context for things I think is useful. |
